# [Retracted] miR‑141 inhibits prostatic cancer cell proliferation and migration, and induces cell apoptosis via targeting of RUNX1

**DOI:** 10.3892/or.2023.8679

**Published:** 2023-12-11

**Authors:** Song Xu, Jingping Ge, Zhengyu Zhang, Wenquan Zhou

Oncol Rep 39: 1454–1460, 2018; DOI: 10.3892/or.2018.6209

Following the publication of the above paper, a concerned reader drew to the Editor's attention that a number of plates showing colony formation assay data in Fig. 3A appeared to be overlapping, such that data purportedly showing the results from differently performed experiments may have been derived from the same original source(s); furthermore, certain of the cell-cycle histograms shown in Fig. 4B were duplicated, again where they were intended to have shown results obtained under different experimental conditions. Subsequently, following an independent enquiry in the office, it was also noted that, in Fig. 2B, the data shown for the RUNX1 western blots were apparently the same for both the DU145 and PC-2 cell lines.

Although a corrigendum was requested by the authors, given the extent of the errors that were identified with respect to the compilation of as many as three of the figures in this paper, the Editor of *Oncology Reports* has decided that this article should be retracted from the publication owing to a lack of overall confidence in the presented data. The authors were asked for an explanation to account for these concerns, but the Editorial Office did not receive a reply. The Editor apologizes to the readership for any inconvenience that might result from the retraction of this article.

